# Dermal Hemorrhage: A Clue to Lichen Sclerosus et Atrophicus

**DOI:** 10.7759/cureus.9343

**Published:** 2020-07-22

**Authors:** Michael Phan, Emily Sou, Ghadah Al Sannaa, Melissa Erwin, Ramon Sanchez

**Affiliations:** 1 Dermatology, University of Texas Medical Branch, Galveston, USA; 2 Dermatology, Medical and Cosmetic Dermatology, El Campo, USA

**Keywords:** lichen sclerosus et atrophicus, bullous, hemorrhagic, lsa

## Abstract

Lichen sclerosus et atrophicus (LSA) may present in a rare bullous and hemorrhagic form that is often difficult to recognize both clinically and histopathologically. Clinically, the lesions may be characterized by atrophic and ivory-white sclerotic plaques in both genital and extragenital regions. Histologically, fully developed lesions of LSA are characterized by a thinned, effaced epidermis with interface change, a wide band of hyalinization in the upper dermis, and a lymphohistiocytic infiltrate below the hyalinized area. Extensive vacuolar degeneration weakens the integrity of the dermoepidermal junction, which contributes to the development of marked edema in the papillary dermis and subepidermal vesiculation. With increased fragility of dermal capillaries, hemorrhage can accumulate within the bullae. Recognizing prominent upper dermal hemorrhage as a secondary change may lead to a prompt diagnosis of LSA. We present a case of extragenital LSA that mimics a dermal hemorrhage clinically and histologically in a 71-year-old Caucasian woman.

## Introduction

Lichen sclerosus et atrophicus (LSA) is a chronic inflammatory condition often characterized by atrophic and ivory-white sclerotic plaques in both genital and extragenital regions. Rare variants, such as bullous and hemorrhagic forms of LSA, are often more difficult to recognize both clinically and histopathologically. Histologically, fully developed lesions of LSA are characterized by a thinned, effaced epidermis with interface change, a wide band of hyalinization in the upper dermis, and a lymphohistiocytic infiltrate below the hyalinized area. Importantly, hemorrhagic or bullous changes may be the predominant feature of LSA. Extensive vacuolar degeneration weakens the integrity of the dermoepidermal junction, which contributes to the development of marked edema in the papillary dermis and subepidermal vesiculation. With increased fragility of dermal capillaries, hemorrhage can accumulate within the bullae [1].

Recognizing prominent upper dermal hemorrhage as a secondary change may lead to a prompt diagnosis of LSA. We present a case of extragenital LSA that mimics a dermal hemorrhage clinically and histologically in a 71-year-old Caucasian woman, highlighting this diagnostic pitfall of LSA.

## Case presentation

A 71-year-old Caucasian female patient with no prior history of skin cancer presented with a red, painful, and enlarging plaque on her right breast for several weeks. This lesion has not been treated in the past and she denies genital lesions.

**Figure 1:**
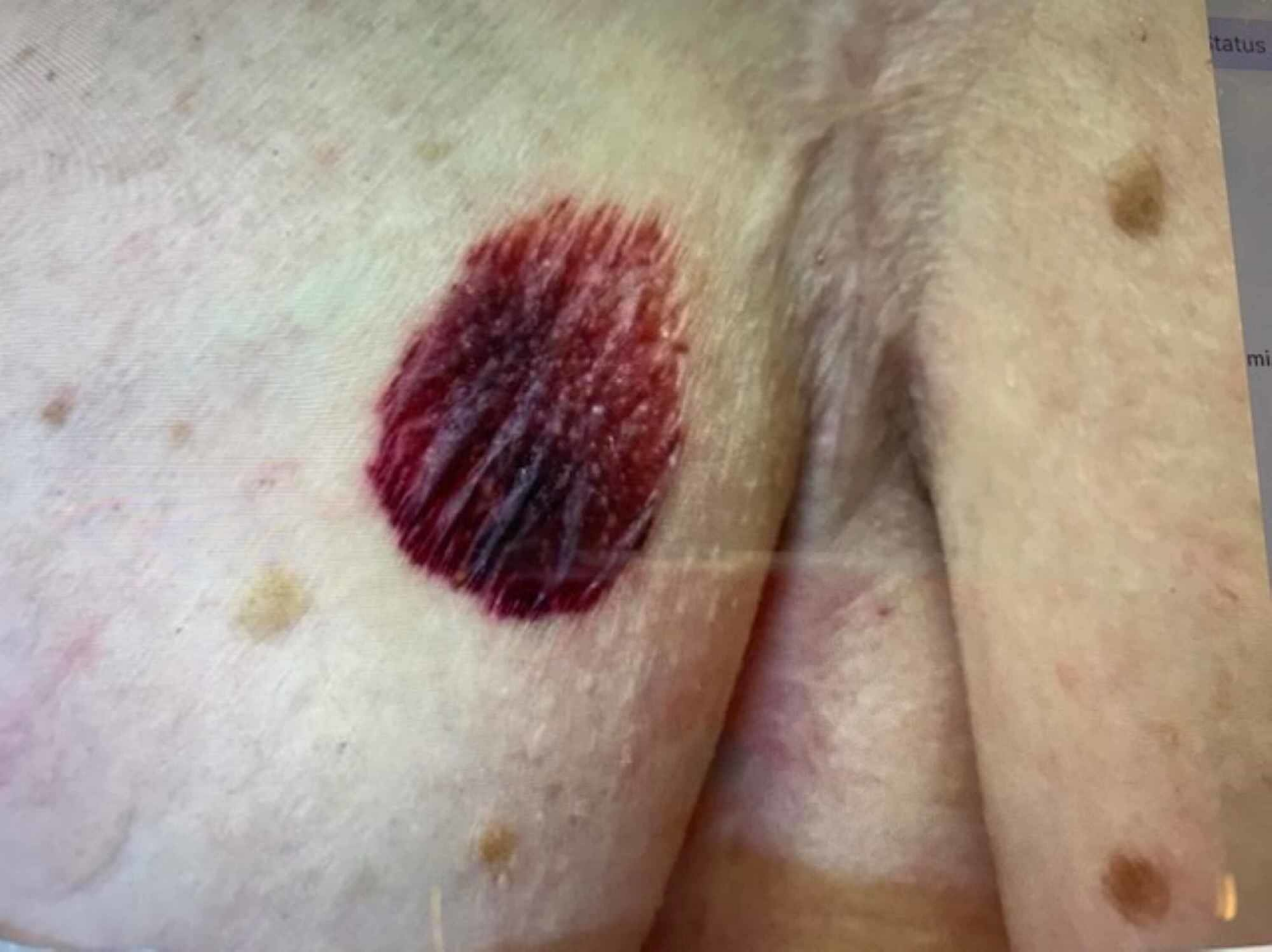
Clinical image of right medial breast demonstrating a well-demarcated fragile hemorrhagic plaque with thin cigarette paper-like texture, with surrounding atrophic skin.

**Figure 2:**
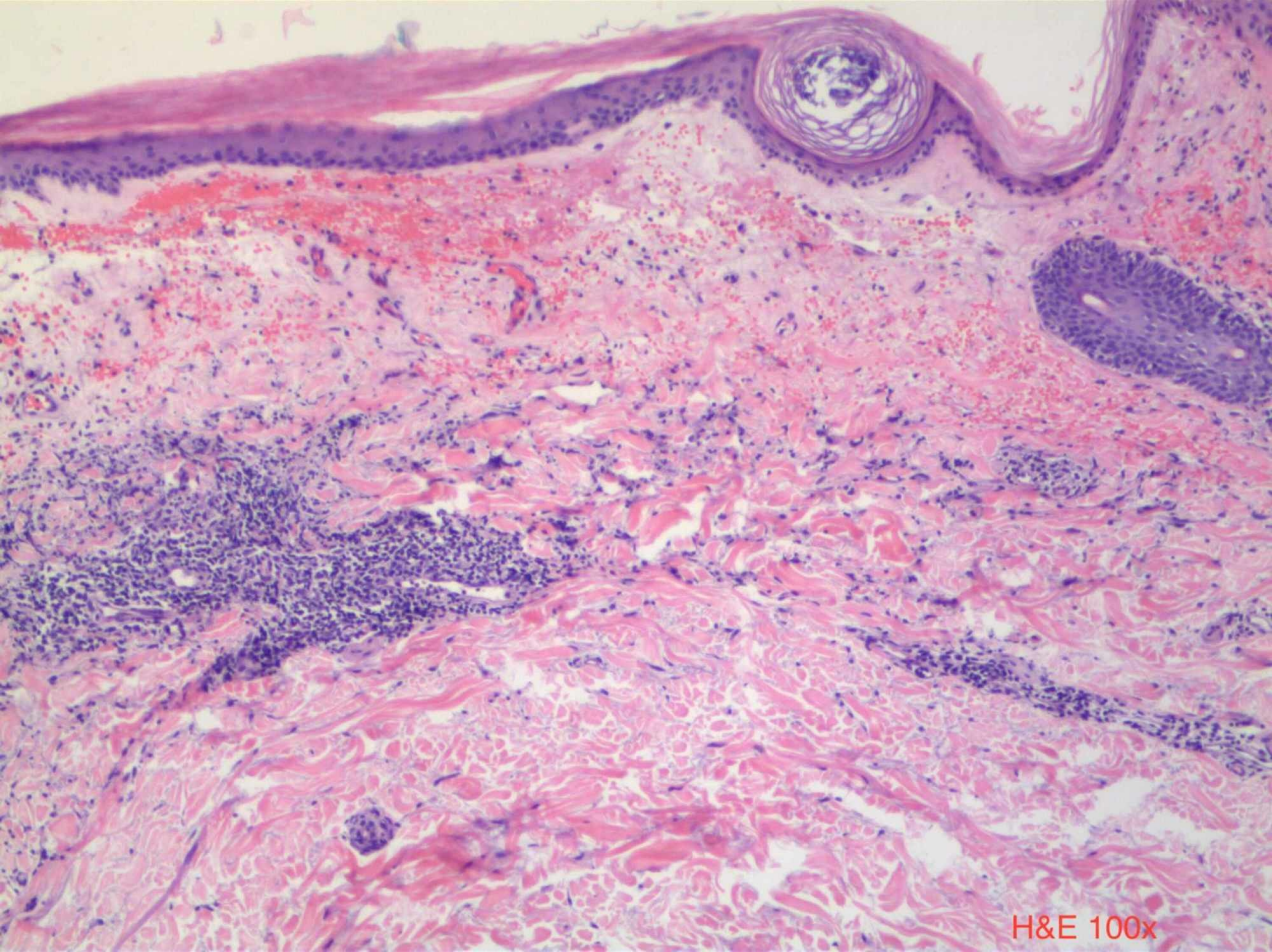
H&E ×100 histology demonstrating hyperkeratosis, epidermal atrophy, papillary dermal edema, and band-like lymphocytic infiltrates directly below. Notice the subepidermal vesiculation and homogenization of the papillary dermis surrounding the hemorrhage.

Physical exam revealed a hemorrhagic plaque with subtle surrounding atrophic changes on her right medial breast (Figure 1). Punch biopsy of the plaque revealed hyperkeratosis with epidermal atrophy, prominent papillary dermal edema with homogenization, and extensive hemorrhage at the dermoepidermal junction, suggestive of LSA (Figure 2). Unfortunately, the patient was lost to follow-up and did not receive treatment.

## Discussion

Pathogenesis

Bullous/hemorrhagic LSA is a severe and rare variant of LSA. This variant of LSA stems from prominent hydropic degeneration of the basal membrane. This feature is also closely associated with marked papillary dermal edema and even with subepidermal vesiculation and hemorrhage. Although the etiology of bullous LSA is unknown, loss of androgen receptor expression, random inactivation of the androgen receptor gene, autoimmunity with type II diabetes mellitus, and Borrelia infection are considered possible causes [1,2]. The extragenital form of LSA occurs in approximately 20% of the patients, and the isolated extragenital lichen sclerosus being even rarer tends to develop unusual clinical presentation, such as the development of vesiculobullous lesions [3]. Multiple authors have reported cases of extragenital of LSA trunk, neck, proximal extremities of arms, scalp, and even orofacial involvement [4-7]. Patients with extragenital hemorrhagic and/or bullous LSA can either present with localized isolated plaques or more extensive lesions with the latter being more difficult to treat. Bullous and non-bullous extragenital LSA have also been reported in association with idiopathic morphea as well as radiation-induced morphea [8,9]. However, overlap between morphea and LSA is unlikely in our patient due to different histopathological features. Morphea is characterized by sclerosis of the reticular dermis, swollen collagen fibers, a perivascular infiltrate, and a loss of adnexal structures. LSA is characterized by follicular plugs and a lichenoid infiltrate in the papillary dermis. Additionally, elastic fibers are normal in morphea but absent in LSA [10].

Diagnosis

Dermascopy of extragenital LSA may reveal scales, keratotic plugs, chrysalis structures, and erosions [11]. Other unusual accompanied features that have been previously described with LSA include telangiectasia, purpura, angiokeratoma-like lesions, and lymphangiectasis [4]. Differential diagnosis of bullous LSA includes bullous lupus erythematosus, bullous cicatricial pemphigoid, bullous scleroderma, bullous lichen planus, trauma, and senile purpura [3]. Recognizing that LSA can, in rare occasions, exhibit prominent upper dermal hemorrhage is essential in making the proper diagnosis. Isolated hemorrhagic plaques and/or bullous lesions may appear prior to the appearance of the more typical plaques of LSA, which can confound clinical diagnosis. Histopathological analysis helps differentiate LSA from other clinically mimicking diseases. Because LSA affected lesions have a long-term risk of squamous cell carcinoma development, close patient follow-up is warranted [12]. 

Management

There is no definitive treatment for bullous LSA, and no randomized controlled trials have evaluated treatment of extragenital LSA [1,12]. Numerous therapies have been used for LSA, including topical and systemic corticosteroids, topical estrogen and testosterone containing ointments, retinoids, tacrolimus, and psoralen plus ultraviolet. However, many cases of bullous LSA may be refractory to first-line treatment consisting of topical corticosteroids. Kreuter et al. reported that patients with refractory generalized extragenital lichen sclerosus benefited from pulsed high-dose corticosteroids combined with low-dose methotrexate treatment [13]. Di Silverio and Serri reported marked improvement in a patient with generalized bullous and hemorrhagic LSA that was treated with adrenocorticotropic hormone [14]. Patients with vulvar LSA refractory to ultrapotent topical corticosteroid have been reported to achieve remission after undergoing fractional carbon dioxide laser with resurfacing or ablative settings [15]. Further studies are needed to determine an effective treatment option for the bullous variant of LSA.

The clinical presentation during resolution of LSA varies from case to case. Bullous lesions of LSA may involute leaving a hyperkeratotic plaque that often tend to persist and are refractory to usual therapies, such as topical corticosteroid therapy and doxycycline [1]. Lima et al. reported a case of hemorrhagic bullous lesion remission with hyperkeratotic lesions after two months of treatment with high-potency topical corticosteroids [3]. Khatu and Vasani reported a patient whose bullous lesions healed with post-inflammatory hypopigmentation and scarring following a short course of oral corticosteroid and topical clobetasol dipropionate 0.05% cream with tacrolimus ointment (0.1%) [16].

## Conclusions

Extensive bullous/hemorrhagic LSA is a severe and rare variant of LSA. This case of extragenital LSA mimicking dermal hemorrhage both clinically and histologically highlights a diagnostic pitfall. Early recognition of such unique extensively hemorrhagic or bullous presentation of LSA can prevent misdiagnosis and inadequate treatment. Further studies are needed to evaluate treatment options for extragenital LSA.
